# Editorial: Stem Cells and Cardiovascular Diseases

**DOI:** 10.3389/fcell.2022.862482

**Published:** 2022-02-28

**Authors:** Miao Yu, Feng Lan, Ming-Tao Zhao, Lei Ye, Shijun Hu

**Affiliations:** ^1^ Department of Cardiovascular Surgery of the First Affiliated Hospital & Institute for Cardiovascular Science, Collaborative Innovation Center of Hematology, State Key Laboratory of Radiation Medicine and Protection, Suzhou Medical College, Soochow University, Suzhou, China; ^2^ State Key Laboratory of Cardiovascular Disease, Fuwai Hospital, Chinese Academy of Medical Sciences, Shenzhen, China; ^3^ Center for Cardiovascular Research, The Abigail Wexner Research Institute and The Heart Center, Nationwide Children’s Hospital, Columbus, OH, United States; ^4^ Department of Pediatrics, The Ohio State University College of Medicine, Columbus, OH, United States; ^5^ Department of Biomedical Engineering, University of Alabama at Birmingham, Birmingham, AL, United States

**Keywords:** stem cells, cardiovascular diseases, mesenchymal stem cells, pluripotent stem cells, heart

Cardiovascular disease remains the leading cause of death worldwide despite significant advances in our understanding of the disease and its treatment. Consequently, the therapeutic potential of stem cells has stimulated a recent surge of translational research and clinical trials aimed at addressing this challenge. In this Research Topic, an overview regarding stem cells and cardiovascular diseases has been provided through 28 articles contributed by 219 authors. Among which, 2 mini reviews, 6 review articles, and 20 original research papers were included.

Huang et al. summarized the applications of human pluripotent stem cells (hPSCs) in heart disease modeling, cell therapy, and next-generation drug discovery. Elde et al. reviewed cell therapy and paracrine signaling approaches to myocardial regeneration. Lin et al. discussed the fascinating perspectives on using patient-specific induced pluripotent stem cells (iPSCs) and clustered regularly interspaced short palindromic repeats (CRISPR) genome editing to functionally study the genetic and epigenetic determinants of congenital heart disease. Gao et al. summarized the protocols of differentiating hPSCs into cardiovascular cells, highlight their therapeutic applications for the treatment of cardiac diseases in large animal models, and discussed the challenges and limitations in using hPSC-derived cardiac cells for a better clinical application of cardiac cell therapy. Meng et al. summarized the current methods of constructing tissue blood vessels *in vitro* and *in vivo*. Liu et al. reviewed the current dilemmas in stem cell-based therapy for ischemic heart disease, including cell source and cell dosage, delivery method and timing of transplantation, cell tracing and the involved mechanisms. Jiang et al. summarized the genetic background of the most common inherited cardiomyopathy and the works have been done so far using hiPSCs for mechanistic research and symptom relief. Huang et al. reviewed the uses of nanoparticles as contrast agents for tracking mesenchymal stem cells (MSCs) and the challenges of clinical applications of MSCs in the treatment of cardiovascular diseases.

This Research Topic contains a series of original research papers related to stem cells and cardiovascular diseases covering methods establishment, mechanism exploration, and so on.

Two papers focus on gene editing. Qi et al. designed an all-in-one episomal vector that expressed a single guide RNA with an adenine base editor or a cytosine base editor. This system can efficiently introduce disease-specific mutations into hPSCs to display phenotype at the cellular level. Gao et al. characterized BlatCas9 as an alternative tool for mammalian genome editing which preferred a N_4_CNAA protospacer adjacent motif and can be packaged into adeno-associated vector (AAV).

Several groups tried to reveal the underlying mechanisms of cardiovascular diseases using patient-specific iPSC derived cardiomyocytes/endothelial cells, such as Brugada syndrome (Li et al.), Duchenne muscular dystrophy related cardiomyopathy (Li et al.), uremic vasculopathy (Jang et al.), and type 2 diabetes mellitus (Su et al.). By using CRISPR/Cas9 technology, Li et al. created a *RRAD* ^−/−^ H9 cell line to disrupt RAD to reveal mechanisms leading to cardiac hypertrophy.

As MSCs have great research value and broad application prospects in the cardiovascular disease, they have been a hot Research Topic. Yu et al. demonstrated that augmenting tripeptidyl peptidase 1 (TPP1) can improve the therapeutic efficacy of aged MSCs in myocardial infarction with enhanced DNA double-strand break repair through AKT/MRE11 pathway. Meng et al. showed that adiponectin modified bone marrow derived MSCs alleviate cardiac fibrosis via inhibiting TGF-β1/Smad in diabetic rats. Li et al. demonstrated that CD73^+^ adipocytes derived MSCs showed a higher pro-angiogenic paracrine activity and displayed therapeutic efficacy for myocardial infarction therapy. Two independent groups developed similar model to visualize, trace, and quantify MSC based on cell-selective metabolic labeling (Zhang et al. and Du et al.). Briefly, mutant methionyl-tRNA synthetase (MetRS*) could utilize non-canonical amino acid azidonorleucine (ANL) instead of methionine during protein synthesis in MSCs. ANL can be covalently linked to alkyne-conjugated reagents via click chemistry. When incubated with ANL-supplemented media, MetRS* expressing MSCs can be detected. This may be leveraged to understand and improve stem cell therapy.

Besides MSCs, pericardial fluid cells (PFCs), perivascular adipose-derived stem cells (PVASCs), cardiac mesenchymal cells (CMCs), and REX-001 were also mentioned. PFCs exhibited progenitor cell features and could serve as an alternative cell source for myocardial tissue repair, engineering, and reconstruction (Sun et al.). PVASCs were capable of differentiating into multiple cell lineages and can contribute to vascular remodeling. As TBX20 inhibited smooth muscle cell differentiation and promoted endothelial cell differentiation from PVASCs, it could be used to inhibit neointima formation after vascular injury (Jie et al.). CMCs were proved to be effective in improving left ventricle function after myocardial infarction. Compared to atmospheric oxygen tension (21%), culturing CMCs at physiologic oxygen tension (5%) provided superior therapeutic efficacy in promoting cardiac repair *in vivo* (Bolli et al.). REX-001 is a kind of human bone marrow derived cell suspension enriched for mononuclear cells, granulocytes and CD34^+^ cells. REX-001 could enhance blood flow recovery after ischemic injury by inducing functional neovascularization (Rojas-Torres et al.).

As for engineered cardiac tissue, a layer-by-layer fabrication method was employed to produce large and thick cardiac tissue created from hiPSC-derived cardiomyocytes, endothelial cells, and fibroblasts. Layer-by-layer fabrication could be utilized to create prevascularized and functional cardiac tissue constructs for potential therapeutic applications (Pretorius et al.). Human ventricular cardiac anisotropic sheets derived from hiPSCs were used to investigate arrhythmogenicity, electrophysiological, and calcium transient changes induced by hypokalaemia (Gurung et al.).

What’s more, Lin et al. revealed the protective effect of XIN against TNNT2 mutation-induced dilated cardiomyopathy. Yang et al. provided an energy-efficient strategy to promote the structural, metabolic and electrophysiological maturation of human embryonic stem cell-derived cardiomyocytes by intermittent starvation.

In summary, this topic provides a platform to exhibit a number of comprehensive reviews and original articles focusing on stem cell-based therapy for treating and modelling of cardiovascular diseases. We thank all authors for their support to make this topic a success.

